# Arctigenin improves neuropathy via ameliorating apoptosis and modulating autophagy in streptozotocin‐induced diabetic mice

**DOI:** 10.1111/cns.14249

**Published:** 2023-05-11

**Authors:** Zekrayat J. H. Medras, Yasser M. Mostafa, Amal A. M. Ahmed, Norhan M. El‐Sayed

**Affiliations:** ^1^ Ministry of Health Kuwait City Kuwait; ^2^ Department of Pharmacology and Toxicology, Faculty of Pharmacy Suez Canal University Ismailia Egypt; ^3^ Department of Pharmacology & Toxicology, Faculty of Pharmacy Badr University in Cairo Badr Egypt; ^4^ Department of Cytology and Histology, Faculty of Veterinary Medicine Suez Canal University Ismailia Egypt

**Keywords:** diabetic neuropathy, arctigenin, oxidative stress, apoptosis, autophagy

## Abstract

**Background:**

Oxidative stress mediates the pathophysiology of diabetic neuropathy (DN) with activation of apoptotic pathway and reduction of autophagy. Arctigenin (ARC) is a natural lignan isolated from some plants of the Asteraceae family that shows antioxidant property. The present study aimed to explore the mechanistic neuroprotective effect of ARC on animal model for DN.

**Methods:**

DN was induced using streptozotocin (STZ) at a dose of 45 mg/kg, i.p, for five consecutive days and ARC was administered orally (25 or 50 mg) for 3 weeks. The mechanical sensitivity and thermal latency were determined using von Frey and hotplate, respectively. Beclin, p62, and LC3 were detected as markers for autophagy by western blot. Levels of reduced glutathione, lipid peroxides, and activities of catalase and superoxide dismutase were detected as readout for oxidative stress. Apoptotic parameters and histopathological changes were revealed in all experimental groups.

**Results:**

The present study showed deterioration of the function and structure of neurons as a result of hyperglycemia. Oxidative stress and impaired autophagy were observed in diabetic neurons as well as the activation of apoptotic pathway. ARC improved the behavioral and histopathological changes of diabetic mice. ARC combated oxidative stress through diminishing lipid peroxidation and improving the activity of antioxidant enzymes. This was concomitant by reducing the biomarkers of apoptosis. ARC augmented the expression of Beclin and LC3 while it lessened the expression of p62 indicating the activation of autophagy. These findings suggest that ARC can ameliorate DN by combating apoptosis and oxidative stress and improving autophagy.

## Graphical abstract

Histological and behavioral changes marked experimental diabetic neuropathy (DN). Oxidative stress, apoptosis, and impaired autophagy were involved. Arctigenin (ARC) reversed these pathological alterations. ARC exhibited antioxidant and anti‐apoptotic properties. ARC may be suggested to protect against DN.

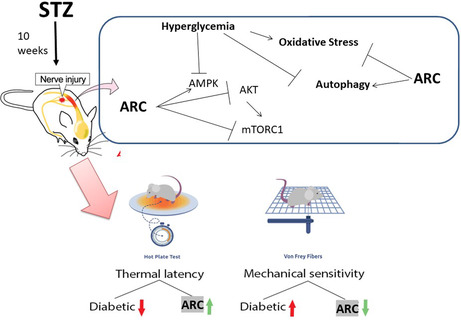

## 1 INTRODUCTION

Diabetic neuropathy (DN) is the mainly identified complication affecting more than half the diabetic patients. It can even affect prediabetic patients who may develop similar neuropathies to diabetic individuals.^1^ Multifactorial pathogenesis is assumed to be implied in DN including inflammatory, nitrosative, and oxidative stress as well as metabolic dysfunction. These can lead to DNA destruction, mitochondrial dysfunction, endoplasmic reticulum stress, and eventually irreversible cellular injury.^2^ Symptoms of DN alter according to the type of sensory fibers implicated. Characteristically, neuropathic pain and dysesthesias are early symptoms for involvement of small fibers. In contrast, patients with affected large fibers experience numbness and loss of pain sensation.^3^

Autophagy is a defensive mechanism that can be activated in response to pathological changes such as the rise of reactive oxygen species (ROS) levels. Eventually, it can maintain normal cell proliferation and growth via the degradation of damaged cells by lysosomal pathway.^4^ Autophagy is a complex multistep cellular process that starts with the formation of phagophore through the combination of some autophagy‐related proteins (Atg). Mammalian target of rapamycin (mTOR) kinase controls this initiation step of autophagy.^5^ Beclin1 (which is also known as mammalian homolog of Atg6) aids to recruit other Atg proteins to the growing phagophore membrane.^6^ The maturation of phagophore to autophagosome involves the recruitment of microtubule‐associated protein light chain (LC3B) (also known as mammalian homolog of Atg8).^6^ The matured autophagosome is then delivered to the lysosomes. The neuropathic disorders including DN are characterized by the existence of intracellular protein aggregates.^7^ The activation of autophagy participates in the clearance of these aggregates thus could be a useful to decrease the symptoms of DN.^8^

Experimental studies on neuronal tissues of diabetic subjects revealed impairment of autophagy.^4^, ^9^, ^10^ Exposure of sera obtained from type 2 diabetic neuropathic patients to neuroblastoma cells caused accumulation of autophagosomes with concomitant increase in LC3 immunoreactivity.^11^ In an animal model of DN, it was observed that there was an dysfunction of autophagy with simultaneous impairment of mitochondria.^12^

Chronic hyperglycemia results in mitochondrial dysfunction with concomitant release of ROS and this leads to bioenergetic crisis as evidenced by reduction of ATP levels.^13^ Consequently, this exacerbates apoptotic pathways marked by reduced levels of Bcl‐2 and activation of caspases in neuronal cells of diabetic subjects.^14^

Arctigenin (ARC) is a bioactive lignan isolated from *Arctium lappa* that shows antioxidant, anti‐inflammatory, and antiviral properties.^15^ These cellular effects are reflected on the ability of ARC to provide a range of pharmacological activities including anti‐tumor, neuroprotection and stamina enhancement. ARC was shown to exert a neuroprotective effect in animal model of ischemic stroke through its anti‐inflammatory property.^16^ It was reported that ARC can protect against memory impairment and decrease beta‐amyloid formation and senile plaques in Alzheimer's disease model mice.^17^ In addition, ARC was shown previously to activate autophagy as evidenced by the upregulation of lipidated LC3 that can be considered a marker for autophagosome formation.^18^ It was postulated that ARC‐mediated activation of autophagy was intermediated through the stimulation of AMP‐activated protein kinase (AMPK) and mTOR signaling pathways.^19^

The link between autophagy, AMPK, and mTOR pathways in the pathophysiology of DN requires additional investigation. The current work was designed to search the possible mechanisms involved in the neuroprotective effect of ARC in mice suffering DN‐induced by streptozotocin (STZ). Oxidative stress, autophagy, and mTOR were the main parameters considered in the current research.

## 2 MATERIALS AND METHODS

### 2.1 Animals

Twenty‐four Swiss albino male mice, weighing 20–25 g and aged 8 weeks old, were used in the current study. Animals were purchased from Nile Co. for pharmaceutical and chemical industries (Egypt). Mice were housed in polypropylene cages with dimensions; 50(L) × 30(W) × 30(H) with wooden bedding in the animal house. The temperature was maintained at 25°C and the animals were kept at 12‐h dark and light cycles. This study was carried out in accordance with the *Guide for the Care and Use of Laboratory Animals* and approved by ethical committee at Faculty of Pharmacy, Suez Canal University (approval no.202012PHDA_2_).

### 2.2 Chemicals

Streptozotocin (STZ) and Arctigenin (ARC) were bought from Sigma Aldrich and Cayman Chemical Company, respectively. ARC was freshly dissolved in 0.5% carboxymethyl cellulose sodium salt solution before its oral treatment.

### 2.3 Induction of diabetes mellitus

To induce type 1 diabetes mellitus, mice were fasted a night before their injection with STZ at a dose (45 mg/kg/day, i.p.)^20^, ^21^ freshly thawed in citrate buffer (pH 4.5) for five successive days. During these 5 days, animals were accessed to normal diet and 10% sucrose water. Afterward, the sucrose solution was substituted with tape water.^20^ A week following the administration of STZ, a blood sample was withdrawn from tail vein to determine glucose concentration. Mice were counted as diabetic and participated in the current study with blood glucose concentration > 250 mg/dL.

### 2.4 Experimental design

Diabetic animals were tested for peripheral DN after 6 weeks of confirmation of hyperglycemic status using von Frey filaments and hot plate test at 55°C, as test for mechanical sensitivity and thermal hyperalgesia, respectively. Von Frey filaments were obtained from Stoelting (USA). Briefly, different filaments ranging from 0.16 to 300 g were applied to the plantar surface of the hind paw of each animal. Positive responses like Lifting or biting of the hind paw were recorded. The threshold was calculated by detecting the minimal force that stimulated at least three positive reflexes of five consecutive trials.^21^ The hot plate apparatus (model LE 7406) was purchased from Lsi LETICA, Italy. The temperature of the apparatus was adjusted at 55°C; then, each mouse was placed on the hot surface. The animals were observed, and the time was recoded when the animals lick their paw or jump off the surface. To prevent tissue damage, a cutoff time of 45 s was considered.^22^

**FIGURE 1:**
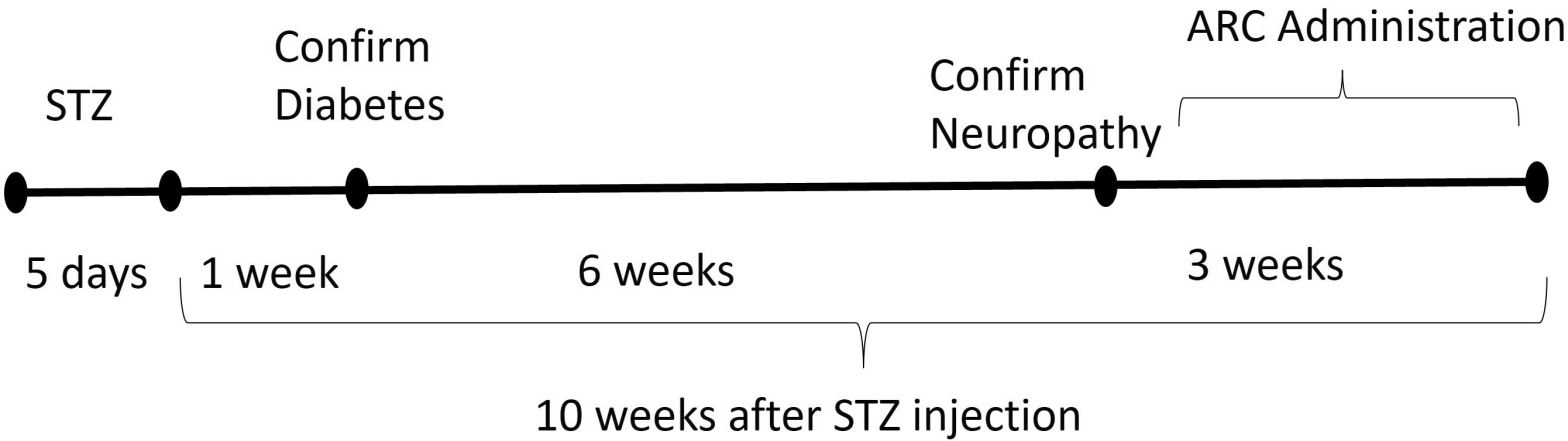
A summary of study design.

After verification of incidence of DN, animals were arbitrarily divided into four experimental groups (six mice each): Group 1, normal control group administered saline; Group 2, diabetic group administered saline; Groups 3 and 4, diabetic mice administered oral doses of ARC (25 or 50 mg/kg) daily for 3 weeks. The selection of ARC doses was based on previous studies.^23^, ^24^ The experimental design is summarized in Figure 1.

After 3 weeks, the behavior was finally assessed by von Frey filaments and hot plate test as previously described.^21^ Mice euthanasia was performed by cervical dislocation under deep anesthesia using ketamine and xylazine at a dose of 87.5 mg/kg and 12.5 mg/kg i.p., respectively. The spinal cord was dissected from each animal and fixed in 10% neutral buffered formalin for use in histopathological and immunohistochemistry staining. The sciatic nerves were isolated and kept at −80°C for the subsequent use for biochemical analysis.

### 2.5 Light microscopic examination

Thoracic and lumbar part of spinal cord tissues were dehydrated in ascending grades of ethanol then exposed to xylol before embedding in paraffin wax. They were sectioned at 4–6 μm thick sections. Microscopic sections were prepared by staining with hematoxylin and eosin (H&E). Slides were analyzed by a blinded researcher without awareness of any information on the experimental groups. The severity of degeneration of spinal cord specimens was scored from 0 to 5 as follows (absent, mild, moderate, severe, very severe, extremely damaged).

### 2.6 Immunohistochemistry

Sections from the spinal cord (5 μm) from each animal was deparaffinized for immunohistochemical examination of Bax and TNF alpha. For antigen retrieval, they were incubated in citrate buffer (adjusted pH = 6) in a microwave. The endogenous peroxidases activity was quenched by using 0.3 % hydrogen peroxide. Vectastain rabbit blocking reagent was applied to prevent nonspecific binding. Recombinant Anti‐Bax antibody [E63] (Cat #ab32503; Abcam) and mouse monoclonal to TNF alpha [52B83] (Cat# ab1793; Abcam) were used as primary antibodies with a dilution 1:100 and incubated overnight.

Secondary antibodies were then applied to each slide for an hour. By using avidin–biotin complex (ABC kit; Vector Laboratories), the bindings of the antibody were visualized. After washing, ABC reagent containing the 3,3′ diaminobenzidine peroxidase enzyme substrate was applied for 5 min. To enhance the nuclear staining, sections were counterstained with hematoxylin. All steps were performed following the instructions of Vectastain Elite ABC Kit (Rabbit IgG, Cat# PK‐6100; Vector Laboratories). Images were taken using a digital camera (Olympus Dp25, Japan). The percent of immunostained area with Bax or TNF alpha was determined using the ImageJ software developed by the National Institute of Health.

### 2.7 Determination of oxidative stress markers

For assessment of oxidative stress, part of sciatic nerve was ice‐cooled and homogenized in 10% phosphate buffer (pH 7.4) using a Teflon homogenizer (Glass Col Homogenizer System). The homogenates were then centrifuged at 3000 ***g*** for 15 min at 4°C. The supernatants were used for the determination of malondialdehyde (MDA) and glutathione (GSH) in addition to the activities of the antioxidant enzymes catalase and superoxide dismutase (SOD). Commercial colorimetric kits for MDA (Cat. No. MD 25 29), reduced GSH (Cat. No. GR 25 11), SOD (Cat. No. SD2520), and catalase (Cat. No. CA 2516) were purchased from Biodiagnostic, Dokki, Egypt. Briefly, levels of MDA were measured using a colorimetric assay based on their reaction with thiobarbituric acid since the resultant color was detected spectrophotometrically at 534 nm. The amount of reduced GSH was quantified depending on its capability to reduce of 5,5′ dithiobis (2‐nitrobenzoic acid). The resulting chromogen is directly proportional to GSH level, and its absorbance can be observed spectrophotometrically at 405 nm.

The activity of SOD was determined based on its capacity to prevent the phenazine methosulfate‐induced reduction of nitro‐blue tetrazolium dye. The activity of catalase was assessed using a colorimetric scheme that is dependent on its reaction with hydrogen peroxide (H_2_O_2_) as substrate and the subsequent measurement of unconverted H_2_O_2_ using a redox dye. The variation in color intensity at 570 nm is reversely proportional to the catalase activity.

### 2.8 Western blotting analysis

Ice cold RIPA buffer, comprising protease inhibitors to preserve the protein and the biological activities of determined enzymes, was used to lysis portions from the sciatic nerves isolated from different experimental groups. The lysates were taken after centrifugation at 16,000 ***g*** for 15 min at 4°C. The protein contents were measured using the Bradford Protein Assay Kit (SK3041) (BIO BASIC INC, Markham Ontario L3R 8T4 Canada). Equivalent volumes of the lysate containing 20 μg of protein and 2× Laemmli sample buffer were mixed and boiled for 5 min at 95°C to ensure the denaturation of proteins. The mixture was loaded into 12% sodium dodecyl sulfate–polyacrylamide gel and exposed to electrophoresis. The protein was next transmitted to PVDF membranes using a Bio‐Rad Trans‐Blot Turbo unit (Bio‐Rad Laboratories Ltd.). The blots were incubated overnight at 4°C with primary antibodies at 1:1,000 dilution in TBS‐T with 5% non‐fat milk; rabbit monoclonal anti‐Bcl‐2 antibody [Cat#ab182858; Abcam], rabbit monoclonal anti‐Bax antibody [Cat#ab32503; Abcam], rabbit monoclonal anti‐cleaved caspase‐3 antibody [Cat#ab214430; Abcam], rabbit polyclonal LC3B antibody [Cat#2775; Cell Signaling Technology], mouse monoclonal nucleoporin p62 [ Cat# sc‐48389; Santa Cruz Biotechnology], rabbit polyclonal Beclin 1 Antibody [Cat# NB500‐249; Novus Biologicals], rabbit monoclonal anti‐Phospho‐AMPKα (Thr172) [Cat#ab 50081; Cell Signaling Technology], rabbit monoclonal anti‐Phospho‐Akt (Ser473) [Cat#ab 4060; Cell Signaling Technology], and rabbit monoclonal Anti‐mTOR (phospho S2448) antibody [Cat#ab109268; Abcam].

Next day, the blots were washed and kept with HRP‐conjugated secondary antibodies (goat anti‐rabbit IgG HRP l mg Goat mab; Novus Biologicals). Bands were visualized using chemiluminescence (ClarityTM Western ECL substrate— cat#170‐5060; BIO‐RAD) following the manufacturer's instructions. Chemiluminescent signals were taken using a CCD camera‐based imager.

### 2.9 Statistical analyses

Statistical tests were performed using the SPSS program, version 22 (SPSS Inc.). Results were represented as the mean ± SD. Quantitative data were analyzed using one‐way analysis of variance (ANOVA) after assessing the normality by Shapiro–Wilk test followed by Tukey's post hoc multiple‐comparisons test. The differences were believed significant at *p* < 0.05.

## 3 RESULTS

### 3.1 Arctigenin reduced the hyperglycemia

**FIGURE 2:**
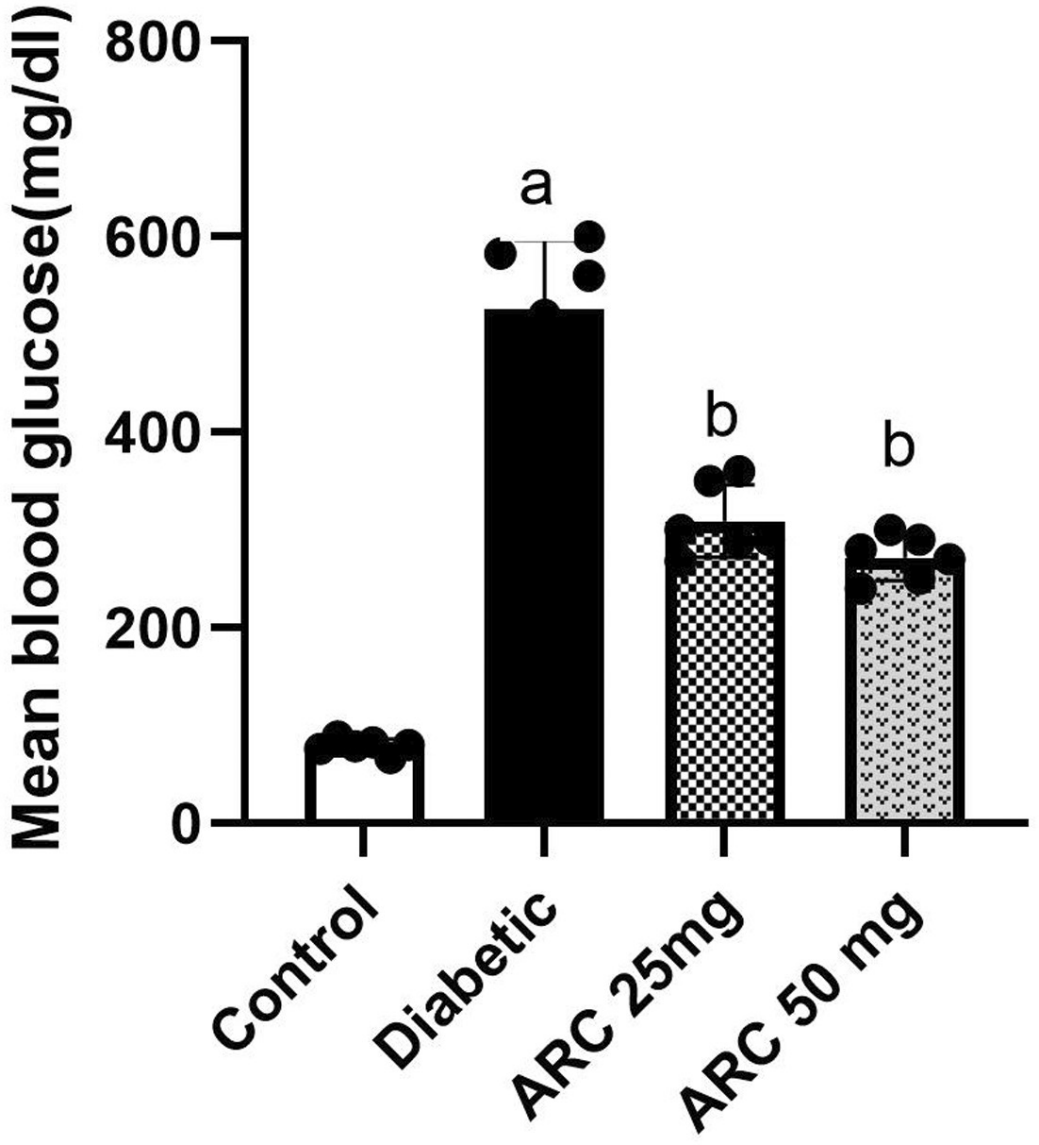
Arctigenin reduced blood glucose levels of STZ‐diabetic mice. Results were expressed as mean ± SD. Superscript symbols indicate a significant difference at *p* ≤ 0.001 using one‐way ANOVA followed by the Tukey's test for multiple comparisons. ^a^Significant differences from normal control. ^b^Significant differences from diabetic mice.

In the present research, the diabetic status was confirmed through the determination of fasting blood glucose levels. Glucose levels significantly elevated in diabetic group compared to normal one to reach 525mg/dL (Figure 2). Administration of ARC orally at two dose levels (25 and 50 mg/kg) for 20 days significantly lessened the hyperglycemic condition to 309 and 270 mg/dL, respectively.

### 3.2 Arctigenin diminished mechanical sensitivity and thermal hyperalgesia

**FIGURE 3:**
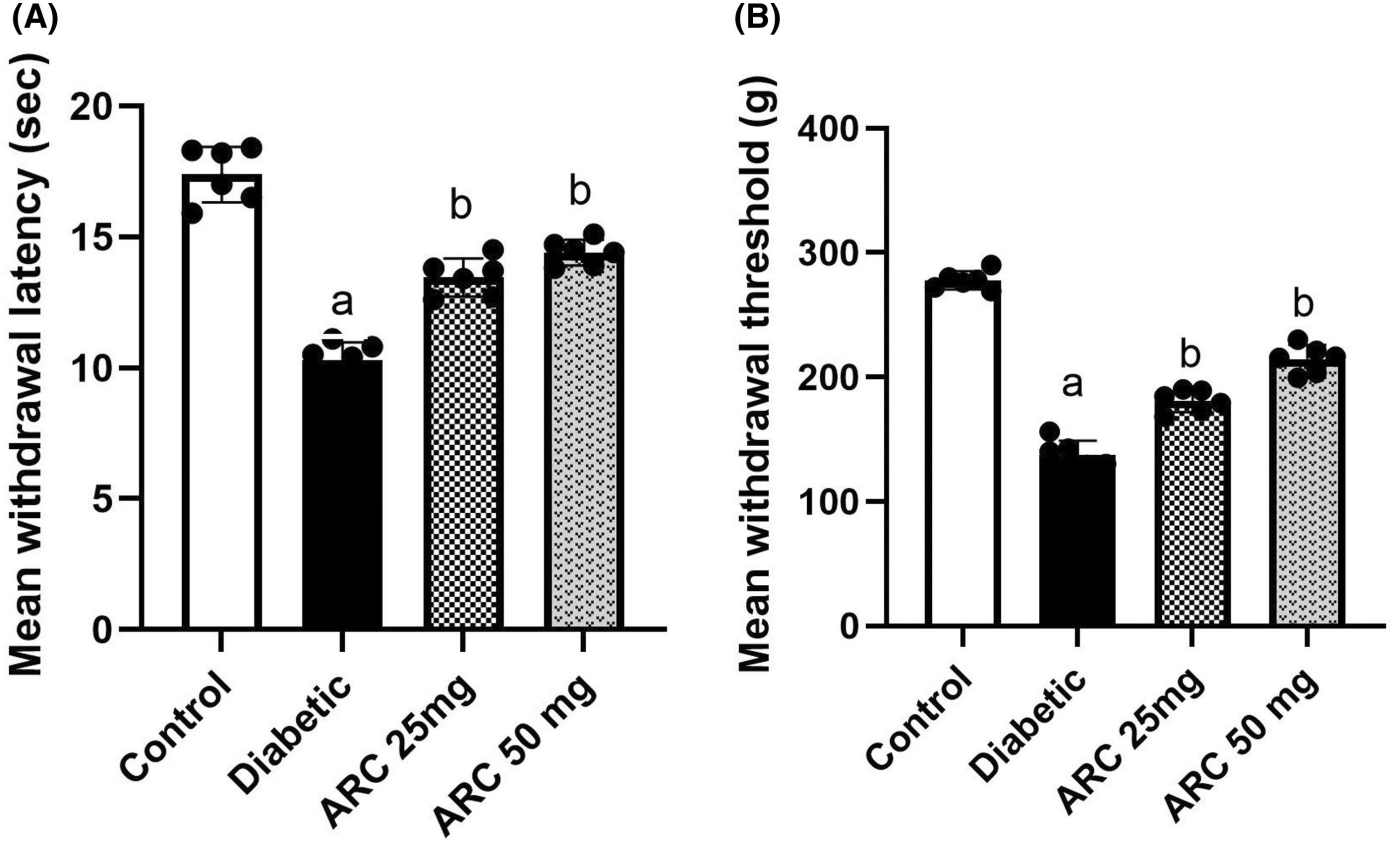
Arctigenin improved the behavioral changes of STZ‐diabetic mice. (A) Effect of arctigenin on thermal latency using the hot plate test. (B) Effect of arctigenin on the mechanical sensitivity using von Frey test. Results were expressed as mean ± SD. Superscript symbols indicate a significant difference at *p* ≤ 0.001 using one‐way ANOVA followed by the Tukey's test for multiple comparisons. ^a^Significant differences from normal control. ^b^Significant differences from diabetic mice.

In von Frey filament test, present results showed a significance drop in the withdrawal threshold of untreated diabetic mice compared to normal mice. However, both groups of mice treated with ARC reversed such decline. Similar results were demonstrated in hot plate test where the untreated diabetic mice showed a significant decrease on the withdrawal latency compared to those of normal mice. Treatment with both doses of ARC reduced the thermal hyperalgesia in diabetic animals (Figure 3).

### 3.3 Arctigenin corrected the histopathological pictures in thoracic and lumbar regions of the spinal cord

**FIGURE 4:**
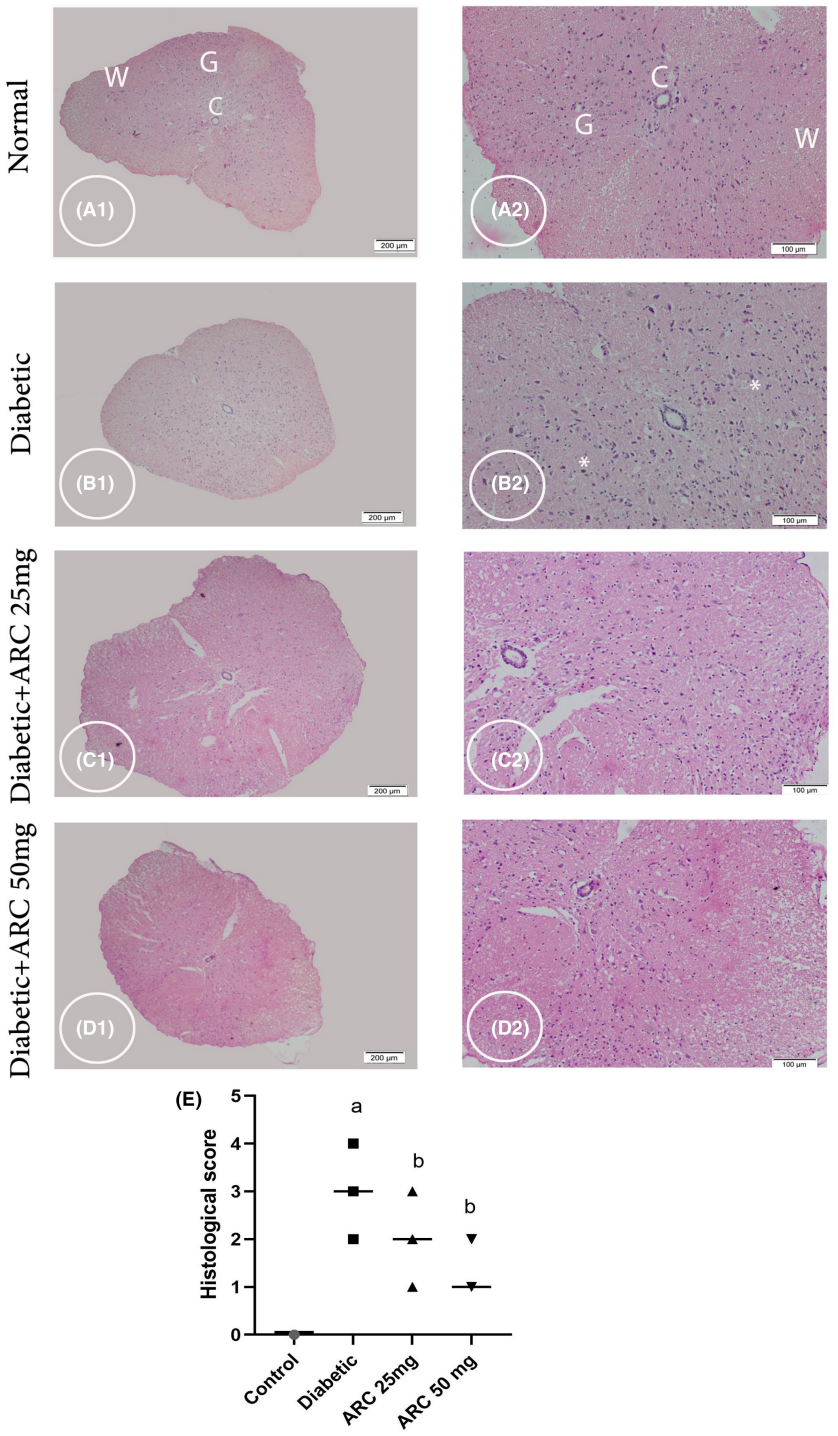
Photomicrograph stained with H&E showing cross sections of thoracic region of the spinal cord (SC). Control group (A1 & A2) displaying normal neurons in the Gray matter (G); note central canal (C) and white matter (W). Diabetic group (B1& B2) displayed shrinkage of some neurons (*) (B2). Diabetic group treated with 25 mg/kg ARC (C1& C2) and diabetic group treated with 50 mg/kg ARC (D1 & D2) displayed an improvement in pathological changes with less shrinkage of neurons. A1, B1, C1, & D1 (scale bar 200 μm) & A2, B2, C2, & D2 higher magnification the gray matter (scale bar 100 μm).

**FIGURE 5:**
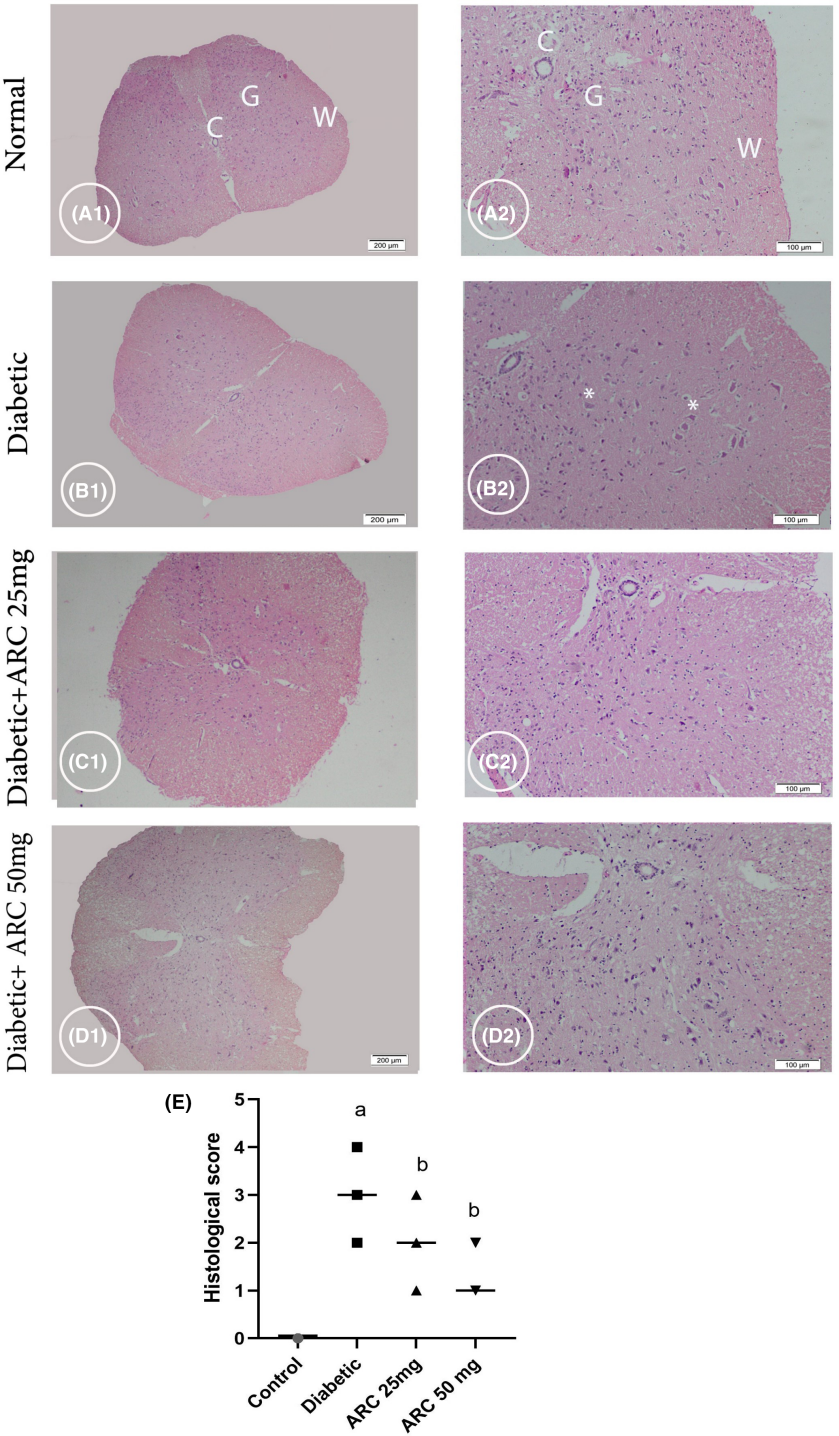
Photomicrograph stained with H&E showing cross section of the lumbar region of the spinal cord. Control group (A1 & A2) displaying normal architecture of central canal (C), gray matter (G), and white matter (W). Diabetic group (B1 & B2) showing shrinkage and faint acidophilia of the neurons (*). Diabetic group treated with 25 mg/kg ARC (C1& C2) and diabetic group treated with 50 mg/kg ARC (D1& D2) showed amelioration of the pathological changes with no apparent shrinkage of the neurons. A1, B1, C1, & D1 (scale bar 200 μm). A2, B2, C2, & D2 (scale bar 100 μm).

Sections from thoracic and lumbar regions from all experimental groups were selected for histopathological examination. Sections from the control healthy groups in both thoracic and lumbar regions demonstrated normal architecture of Gray matter (G), central canal (C), and white matter (W) as showed in Figure 4A1,A2. In the thoracic regions of the diabetic mice, sections exhibited shrinkage in some neurons (Figure 5B1,B2). In addition, sections of the lumbar region isolated from diabetic mice also showed shrinkage and faint acidophilia of the neurons (Figure 5B1,B2). Degeneration of the gray matter was observed in both regions of STZ diabetic groups. However, oral treatment with ARC improved the histopathological picture and ameliorated the pathological changes with no apparent shrinkage of the neurons, especially in the lumbar region (Figure 5C1,C2) and (Figure 5D1,D2).

### 3.4 Arctigenin reduced some biomarkers of apoptosis

**FIGURE 6:**
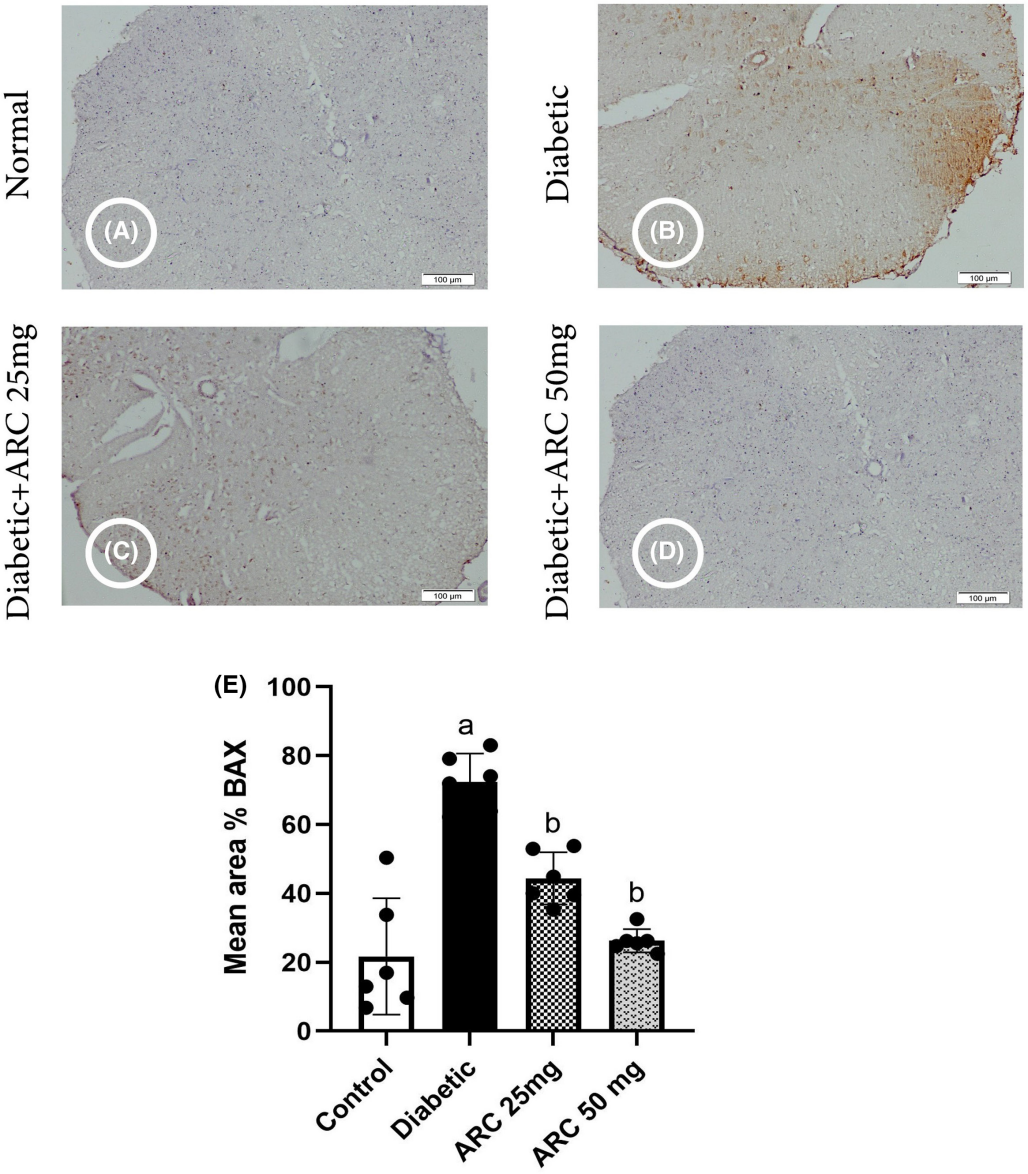
Photomicrographs of the spinal cord immunostained with BAX. (A) Control group, (B) diabetic group, (C) diabetic group treated with 25 mg/kg ARC, (D) diabetic group treated with 50 mg/kg ARC, (E) mean of area % of immunoreaction in spinal cord immunostained with BAX. Results were expressed as mean ± SD. Superscript symbols indicate a significant difference at *p* ≤ 0.05 using one‐way ANOVA followed by the Tukey's test for multiple comparisons. ^a^Significant differences from normal control. ^b^Significant differences from diabetic mice (Scale bar 100 μm).

Upon assessment of the spinal cord immunostained with Bax, it was revealed that normal control mice displayed weakly positive staining reactivity of cells (Figure 6A). However, intensely positive staining reactivity of the cells isolated from STZ‐induced diabetic mice was shown (Figure 6B). Sections from the spinal cord of ARC‐treated mice exhibited weakly positive staining reactivity and weak immunostaining of cells lying beneath (Figures 5D and 6C). Mean % area of apoptotic cells immunostained with Bax in diabetic mice was extensively risen compared to normal and ARC‐treated mice (Figure 6E).

**FIGURE 7:**
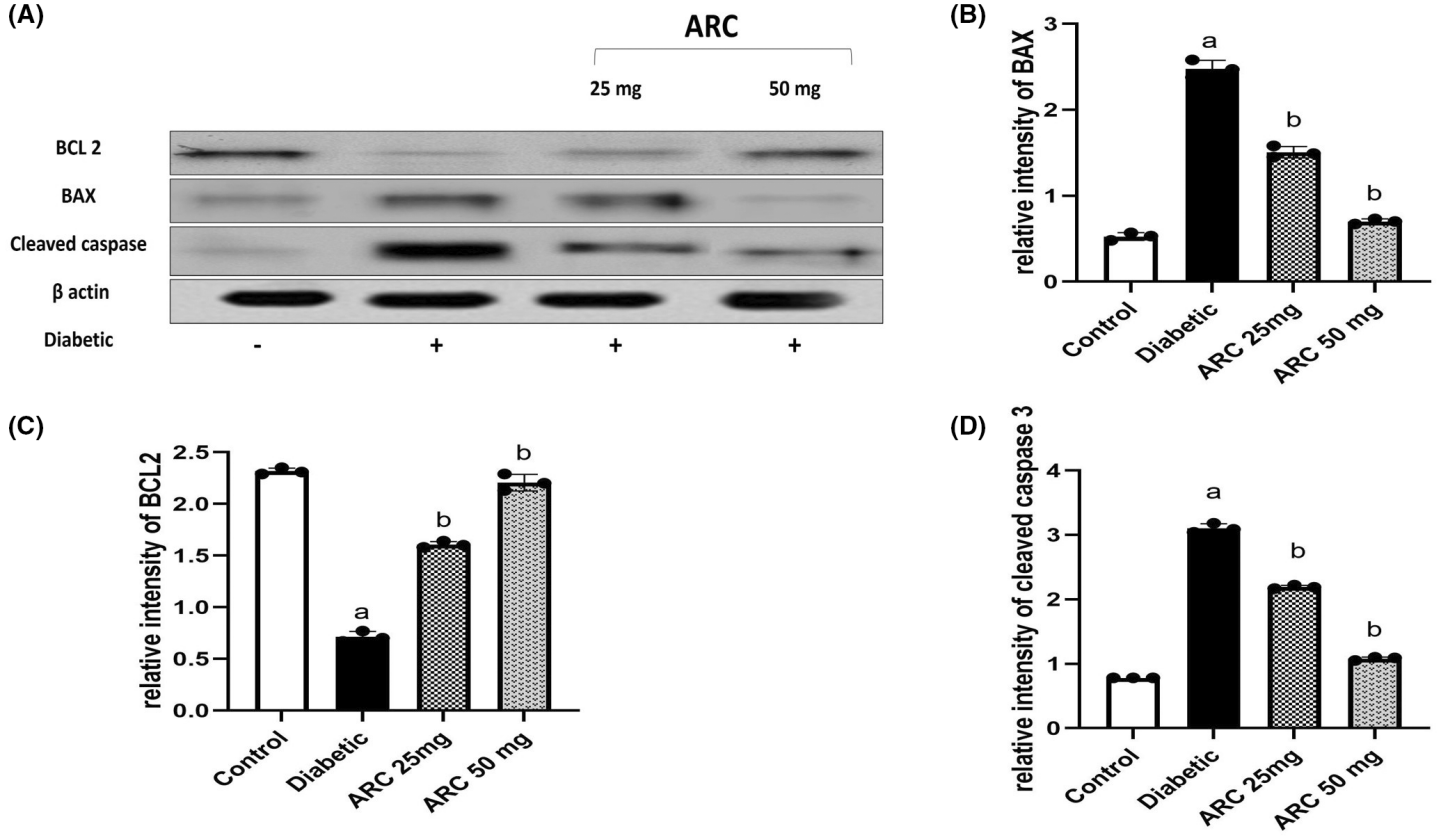
Arctigenin downregulated BAX and cleaved caspase while increased levels of Bcl‐2 in STZ‐diabetic mice. (A) Western blotting was performed using antisera against Bcl‐2, BAX and cleaved caspase β‐actin served as a control for loading. (B) Quantitative analysis of Bcl‐2 normalized to β‐actin. (C) Quantitative analysis of BAX normalized to β‐actin. (D) Quantified analysis of cleaved caspase normalized to β‐actin. Results were expressed as means ± SD. Analysis of quantitative variables was performed using one‐way ANOVA followed by Tukey's post hoc test with *p* < 0.05 considered statistically significant. ^a^Significant difference from normal; ^b^Significant difference from diabetic mice.

Similarly, the expression levels of Bax and cleaved caspase 3 proteins were significantly high in sciatic nerves of diabetic mice compared with the normal mice (Figure 7C,D). In contrast, treatment with ARC significantly reduced both Bax and cleaved caspase 3 (Figure 7C,D); where the high dose (50 mg/kg) of ARC showed a significant reduction of the expression of both apoptotic markers compared to the lower ARC dose (25mg/kg). Simultaneous with the upregulation of Bax (proapoptotic), the expression of Bcl‐2 (anti‐apoptotic) was reduced in the sciatic nerves of diabetic group (Figure 7B). Treatment with ARC (50 mg/kg) restored the normal expression of Bcl‐2 as shown in Figure 7B.

### 3.5 Arctigenin ameliorated oxidative stress in STZ‐diabetic mice

Table 1 represents the changes in some biomarkers of oxidative stress including MDA, GSH concentrations, and the activities of catalase and SOD as a result of induction of diabetes by STZ. Sciatic nerves of diabetic mice showed an increase of MDA content while the levels of reduced GSH were notably lower compared to normal control mice. Treatment with ARC inhibited the rise of MDA concentrations and reversed the decline of active GSH in a dose‐dependent manner.

**TABLE 1:** Effects of ARC on some biochemical indicators of oxidative stress, namely MDA and GSH levels, catalase, and SOD activity.

	Normal control	Diabetic	ARC 25mg	ARC 50 mg
MDA (nmol/mg protein)	2.7 ± 0.4	7.8 ± 0.5^a^	3.4 ± 0.5^b^	2.6 ± 0.5^b^
GSH (μmol/mg protein)	103.1 ± 20.1	55.1 ± 7.3^a^	88.6 ± 11.7^b^	94.1 ± 10.3^b^
Catalase (U/mg Protein)	83.6 ± 16.7	42.1 ± 7.6^a^	74.1 ± 9.7^b^	80.3 ± 8.1^b^
SOD (U/mg protein)	56.5 ± 8.5	25.8 ± 7.1^a^	51.1 ± 7.3^b^	53.5 ± 7.1^b^

*Note*: Values are expressed as means ± SD. Superscript letters indicate a significant difference at *p* ≤ 0.05 using one‐way ANOVA followed by the Bonferroni's test for multiple comparisons.

^a^ Significant differences from normal control.

^b^ Significant differences from diabetic mice.

The current study pointed out the significant decrease in the activities of catalase and SOD upon induction of diabetes compared to the normal control group. However, a marked increase in the activities of both enzymes was demonstrated after the administration of ARC to STZ diabetic mice (Table 1). ARC administrated with a dose of 50 mg/kg was able to restore the normal activities of catalase and SOD.

### 3.6 Arctigenin reduced the immunohistochemical staining of tumor necrosis factor alpha

**FIGURE 8:**
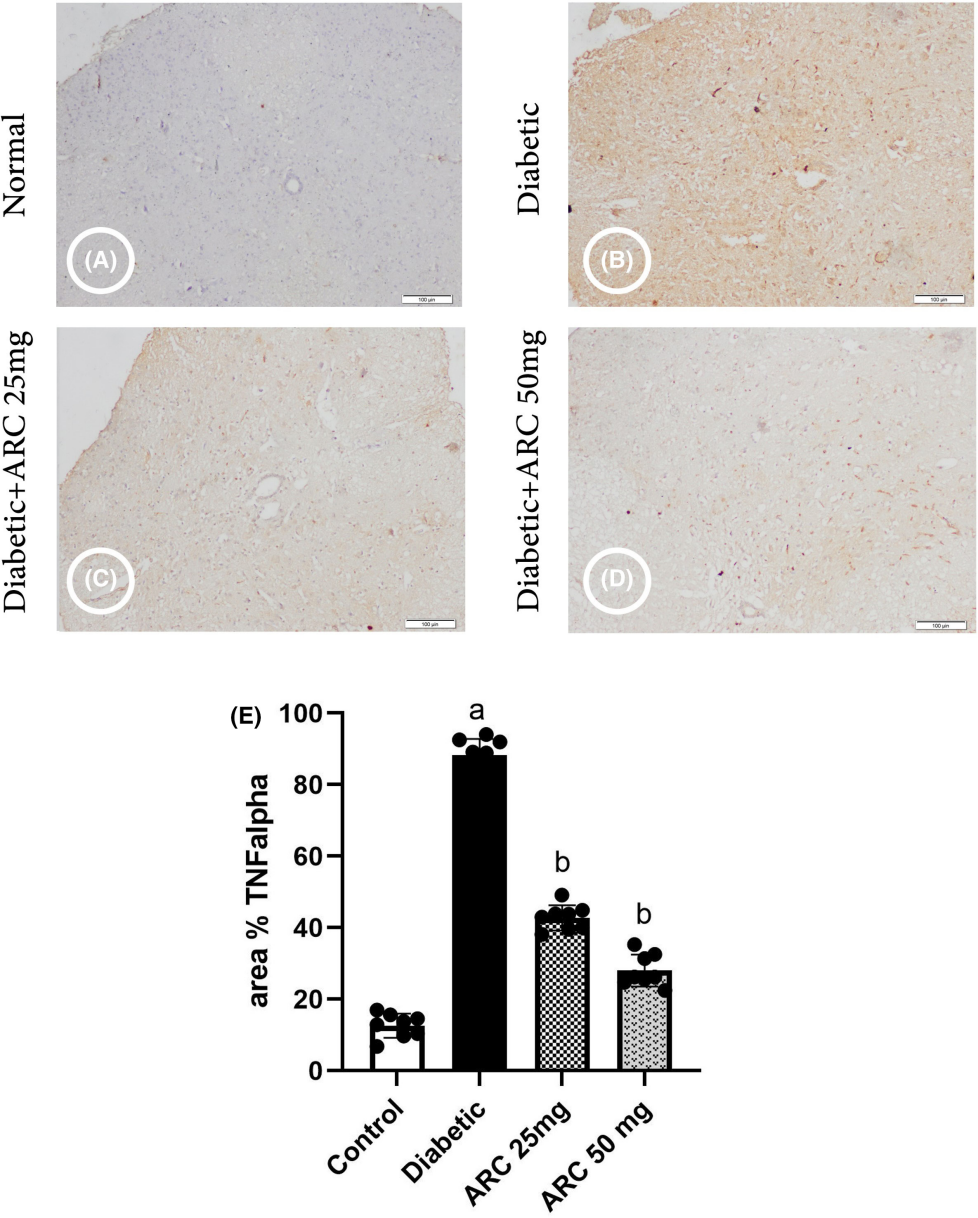
Photomicrographs of the spinal cord immunostained with tumor necrosis factor alpha. (A) Control group, (B) diabetic group, (C) diabetic group treated with 25 mg/kg ARC, (D) diabetic group treated with 50 mg/kg ARC, and (E) mean of area % of immunoreaction in spinal cord immunostained with TNF alpha. Results were expressed as mean ± SD. Superscript symbols indicate a significant difference at *p* ≤ 0.05 using one‐way ANOVA followed by the Tukey's test for multiple comparisons. ^a^Significant differences from normal control. ^b^Significant differences from diabetic mice (Scale bar 100 μm).

Figure 8 shows the photomicrographs of the spinal cord immunostained with TNF alpha. Diabetic group exhibited a significant increase in the immunostaining intensity of TNF alpha indicating the occurrence of inflammation. Treatment with either doses of ARC (25 or 50 mg/kg) reduced significantly the area of spinal cord immunostained with TNF alpha.

### 3.7 Arctigenin activated AMPK and downregulated AKT/mTOR pathway

**FIGURE 9:**
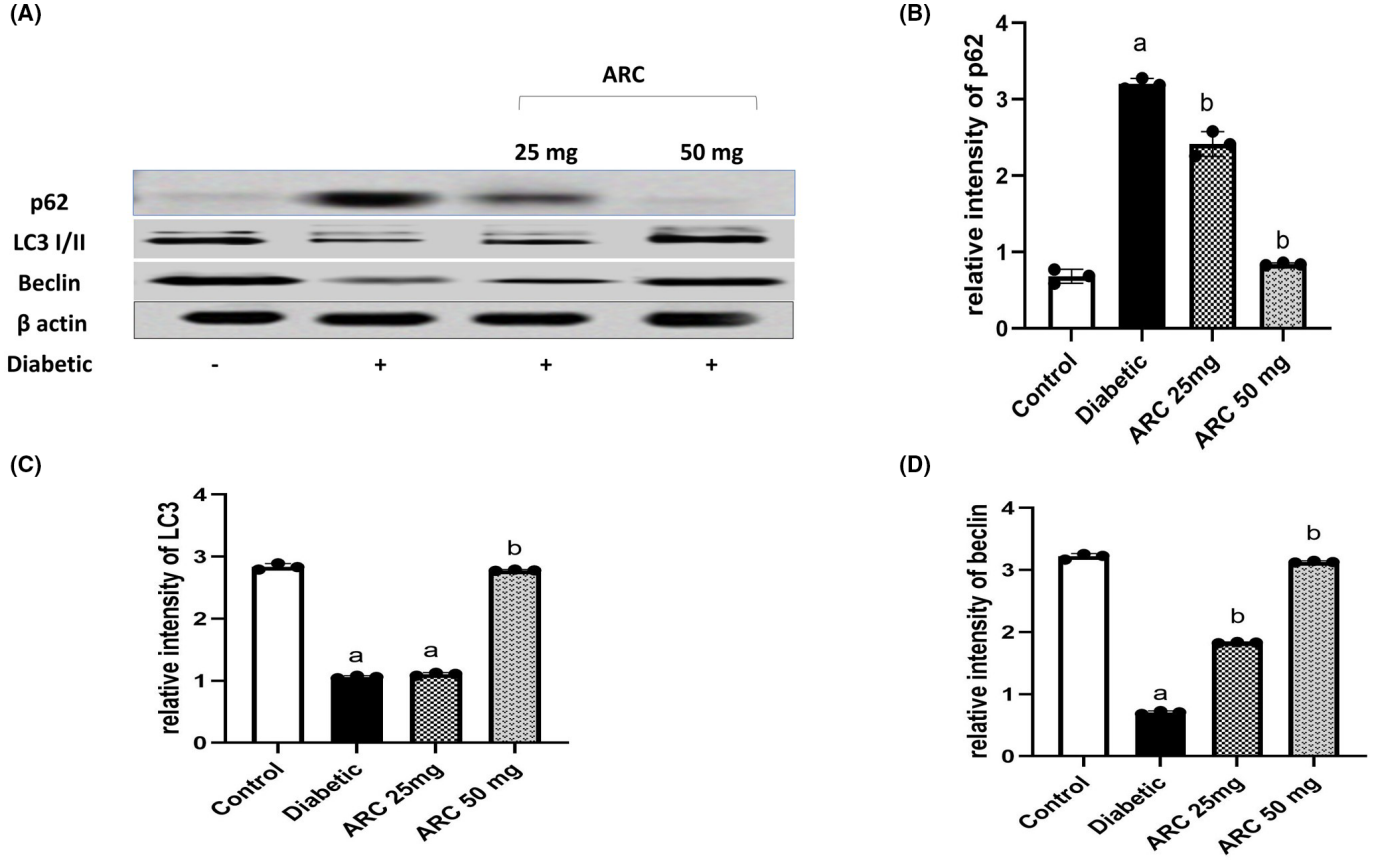
Arctigenin activated AMPK while reduced the expression of P‐AKT/mTOR. (A) Western blotting was performed using antisera against AMPK, P‐AKT, P‐mTOR, β‐actin served as a control for loading. (B) Quantitative analysis of p‐AMPK normalized to β‐actin. (C) Quantitative analysis of P‐AKT normalized to β‐actin (D) Quantified analysis of P‐mTOR normalized to β‐actin. Results were expressed as means ± SD. Analysis of quantitative variables was performed using one‐way ANOVA followed by Tukey's post hoc test with *p* < 0.05 considered statistically significant. ^a^Significant difference from normal; ^b^Significant difference from diabetic mice.

Figure 9 depicts the protein expressions of p‐AMPK α (Thr172), p‐AKT (Ser473), and p‐mTOR in sciatic nerves of different experimental groups. Treatment with ARC significantly activated the AMPK pathway as noted by the increase of its phosphorylation in a dose‐dependent manner. In addition, a marked upregulation of Akt/mTOR pathway was shown in sciatic nerves isolated from STZ‐treated mice as evidenced by the increase of the phosphorylation of AKT at ser473 and that of mTOR at ser2448. Treatment with ARC extensively diminished the protein expression of AKT and mTOR especially at the higher dose (50 mg/kg) that showed a similar picture to the normal control mice.

### 3.8 Arctigenin restored the activity of autophagy

**FIGURE 10:**
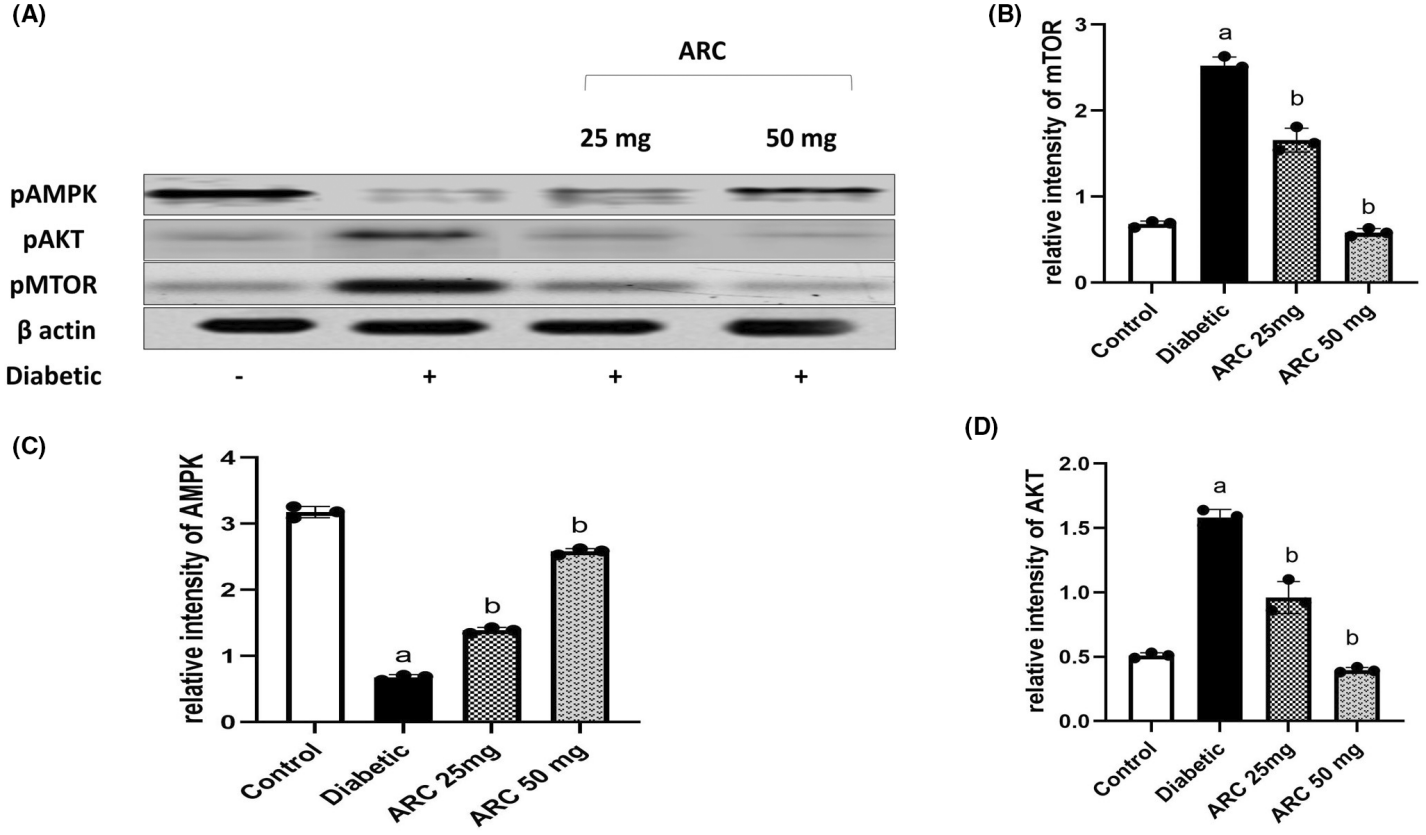
Arctigenin activated autophagy pathway (A) Western blotting was performed using antisera against Beclin, LC3 I/II, and p62, β‐actin served as a control for loading. (B) Quantitative analysis of Beclin normalized to β‐actin. (C) Quantitative analysis of LC3I/II normalized to β‐actin. (D) Quantified analysis of p62 normalized to β‐actin. Results were expressed as means ± SD. Analysis of quantitative variables was performed using one‐way ANOVA followed by Tukey's post hoc test with *p* < 0.05 considered statistically significant. ^a^Significant difference from normal; ^b^Significant difference from diabetic mice.

Induction of diabetes by STZ impaired autophagy pathway as evidenced by the marked decrease of LC3‐I and II (a key biomarker of autophagy) in sciatic nerves of diabetic mice compared to normal mice. Consistently, lower levels of Beclin were observed in the sciatic nerves of diabetic mice while the levels of p62 were higher in the same tissues compared to normal mice (Figure 10). Interestingly, ARC prevented the impairment of autophagy pathway and treatment with ARC (50 mg/kg) presented a similar picture to normal mice. ARC increased the expression of LC3‐I/II and Beclin while significantly reduced the expression of p62.

## 4 DISCUSSION

Peripheral DN results in various deterioration of the function and the structure of neurons. This was obvious in the present study as persistent hyperglycemia induced by STZ resulted in neurobehavioral impairments as well as significant histopathological changes. Significant degeneration of nerve fibers besides the enlargement of mitochondria are evidence for the axons damage and mitochondrial dysfunction. This was reflected on the assessment of thermal allodynia and mechanical sensitivity where STZ‐treated mice showed significant reduction of thermal latency and mechanical sensitivity. Similarly, it was previously reported that injection of STZ developed hypoalgesia and mechanical insensitivity in mice^25^ suggesting that hyperglycemia can lead to sensory neuropathy. In addition, diabetic mice showed slowing of nerve conduction indicating dysfunction of axons concomitant with reduction of unmyelinated fibers of axon area.^25^

The neurobehavioral deterioration observed in the current study mimics the symptoms experienced by DN patients. Mainly DN is sensory dominant, and patients suffer either positively; paresthesia, allodynia, pain or negatively; hypoalgesia and numbness.^26^ Postmortem study of patients with DN demonstrated infiltrates of mononuclear inflammatory cells with predominant demyelination of axons.^27^ In addition, necrotizing arteritis and microvasculitis were observed in the peripheral nerve biopsies.^27^

The current study revealed that induction of diabetes was associated with significant activation of apoptosis pathway. Diabetic neurons showed reduced levels of Bcl‐2 that can result from either decrease synthesis or enhancement of its proteolysis. This was concomitant with the increase in BAX expression. Another interesting observation was the upregulation of cleaved caspase. Together these results suggest the activation of apoptotic pathway in neurons following the hyperglycemic status. This was reflected on the histopathological impairment in diabetic neurons where there is an increase of degenerated cells.

Multiple mechanisms were suggested to be involved in the pathophysiology of DN including oxidative stress, inflammation, and recently autophagy. Autophagy is a cellular process that encompasses the recycle of damaged organelles and protein in autophagosomes. Autophagy is additionally involved in clearing cells undergone physiological programmed cell death.^28^ Dysregulation of autophagy was reported in the pathogenesis of several diseases including neurodegenerative disorders.^29^, ^30^ In normal cells, autophagy is crucial for survival, growth, and differentiation, and thus, it is an important pathway to help the body cope with stressful conditions.

Chronic hyperglycemia observed in the current study resulted in significant increase in the oxidative stress markers. Oxidative stress can occur as result of the rise of free radicals or the decline in the endogenous antioxidant defense mechanism. In case of diabetes, mainly autoxidation of glucose and its metabolite is the main source of free radicals.^1^, ^31^ The present results highlighted the significant increase in MDA as a readout of lipid oxidation and the decline of antioxidant enzymes; catalase and SOD besides the decease of reduced GSH. Activities of the cytosolic nicotinamide adenosine diphosphate (NADP)–dependent dehydrogenases and NADP malic enzymes are diminished in the hyperglycemic status, and in turn, this can lessen the amount of nicotinamide adenosine diphosphate hydrogen (NADPH) intracellularly.^13^ Typically, NADPH is used by GSH reductase to provide sufficient concentrations of the reduced form of GSH.^13^ This could explain the decline of the active form of GSH observed in specimens of diabetic group. The rise of free radicals causes the increase of mitochondrial permeability transition pore size with simultaneous release of cytochrome c from its store in the mitochondria to the cytoplasm and this activated the apoptotic pathway as remarked in the current study.

Accumulation of degenerated cells and toxic metabolites in DN requires the effective removal of damaged organelles and aggregated protein by a mechanism like autophagy. Autophagy induction can reduce the oxidative stress, enhance ATP production, and lessen apoptosis through Bcl‐2 activation.^32^ There is some evidence of accumulation of autophagosomes upon exposure of sera separated from type 2 DN patients to neuroblastoma cells. This was confirmed by increased LC3II immunoreactivity.^11^ However, over activation under persistent stressful conditions like sustained hyperglycemia and its harmful consequences could lead to impairment of autophagy and trigger the apoptotic pathway. Beclin, LC3, and p62 are major proteins participated in the regulation of autophagy. Beclin is essential for the initiation of autophagy and maturation of autophagy bodies.^32^ LC3 is normally found in the cytoplasm in the form of LC3I, upon activation of autophagy LC3I is converted to LC3II. LC3II is crucial for the formation of autophagosome.^32^ In the current research, the expression levels of both Beclin and LC3 were reduced in the sciatic nerves of diabetic animals. This indicates the inactivation of autophagy pathway and its failure to remove the deleterious toxic metabolites and damaged cells. Similarly, in experimental model of DN, it was demonstrated that there was an impairment of autophagy reflux with concomitant mitochondrial dysfunction.^12^

The serine/threonine kinase (AKT) is a downstream of phosphatidylinositol kinase (PI3K) and is responsible to transmit the signal received by PI3K.^33^ Inflammatory cytokines can activate PI3K.^34^ Diabetic neurons showed an increase in the release of inflammatory cytokines.^35^ This was evidenced in our study by the increase in the percent of spinal cord sections immunostained with TNF alpha. mTOR is a downstream of PI3K/AKT pathway and is involved in activation of various transcription proteins. Autophagy can be induced by inhibition of mTORC that inhibits the formation of autophagosomes.^5^ Several studies have indicated that the activation of PI3K/AKT/mTOR pathway is involved in the occurrence of neuropathic pain.^21^, ^36^ Therefore, there are several drugs and natural products acting to downregulate this pathway were shown to protect against neuropathies.^9^, ^37^, ^38^, ^39^ The current study showed that AKT/mTOR pathway was activated in the specimens isolated from diabetic mice as evidenced by the increased expression of phosphorylated AKT and mTOR.

In the current study, the activity of AMPK was diminished as a result of sustained hyperglycemia and the impairment of bioenergetics. AMPK is a serine/threonine kinase that can be activated by AMP following the rise in the AMP/ATP ratio. This is consistent with a previous study showing a significant decrease of AMPK in dorsal root ganglia neurons isolated from diabetic neurons.^40^ The decline of AMPK activity is another cause for axonal structural deterioration. The axons are mainly susceptible to the decrease of ATP as axonal plasticity generally needs enough supply of energy to undertake continuous turnover.^41^ In normal conditions, AMPK is responsible for the activation of tuberous sclerosis complex 2 (TSC2) that negatively regulates mTOR activation.^42^ Moreover, AMPK can phosphorylate insulin receptor substrate 1 (IRS1) and AKT at their negative regulatory sites which further inactivates mTORC1 signaling pathway.

Arctigenin appeared in this study to exert a neuroprotective effect against STZ‐induced DN. ARC attenuated neuropathic pain experienced by diabetic mice evidenced by enhanced thermal latency and reduced mechanical sensitivity. ARC improved the histopathological changes in different parts of spinal cord and reduced the percentage of apoptotic cells. Administration of ARC significantly reduced blood glucose of hyperglycemic mice. It was previously shown that ARC was able to reduce the activity of α‐glucosidase which is a hydroxylase enzyme that hydrolyzes starch to glucose.^43^ A study in GK rats administered the active constituents of *Fructus Arctii* (mainly arctigenin) revealed a decline of glucagon levels with concomitant increase of insulin levels.^43^ This study suggested that this effect was mediated by the release of glucagon like peptide to produce incretin effect. Another study demonstrated that derivatives of *Fructus Arctii* can activate AMPK to increase adiponectin levels to reverse insulin resistance.^44^

In addition to the glucose‐lowering effect experienced by ARC administration, ARC showed its ability to combat oxidative stress. The present study demonstrated that ARC lessened the production of MDA; a biomarker for lipid peroxidation as well as increased the endogenous active form of glutathione. Besides, ARC restored the activity of the antioxidant enzymes; catalase, and SOD. The antioxidant effect of ARC was previously shown in a model of oxidative stress induced in lungs by lipopolysaccharide.^45^ The antioxidant effect of ARC on diabetic neurons provides an important mechanism in protecting the deleterious effect of chronic hyperglycemia. This was evidenced by the improvement of histopathological picture and the reduction of apoptotic markers.

The current study revealed the ability of ARC to downregulate the activity of mTORC1 in diabetic neurons. Similar effect of ARC on mTORC1 activation was reported in dextran sulfate sodium‐induced colitis mice.^46^ This study suggested that the effect of ARC on mTOR could be explained by its ant‐inflammatory effect and its consequence on reducing the cytokines. In the present study, there was a reduction of the area of spinal cord immunostained with TNF alpha upon the treatment with ARC. Another study showed that the effect of ARC to inhibit the PI3K/AKT/mTOR pathway was mediated by reduction of NF‐kB activation.^47^ Downregulating the PI3K/AKT/mTOR pathway resulted in regulation of autophagy as demonstrated in this study. ARC activated autophagy in diabetic neurons as the protein levels of Beclin and LC3 II were increased. Matching results were observed by ARC in glioma cells where ARC activated autophagia markers and reduced the activity of AKT/mTOR.^48^

To conclude, the present study provided some evidence on the neuroprotective effect of ARC in STZ‐induced DN. ARC exerted antioxidant and antiapoptotic effect with concomitant activation of autophagy and downregulation of AKT/mTOR pathway. These results propose that ARC could be a prospective therapy for DN and that the intake of arctigenin could be an effective supplement for diabetic patients.

## FUNDING INFORMATION

This research did not receive any specific grant from funding agencies in the public, commercial, or not‐for‐profit sectors.

## CONFLICT OF INTEREST STATEMENT

The authors declared that there are no conflicts of interest.

## Supporting information


Figure S1.

## Data Availability

The data that support the findings of this study are available on request from the corresponding author.
