# Supplementary material for: Arctigenin improves neuropathy via ameliorating apoptosis and modulating autophagy in streptozotocin‐induced diabetic mice
Source: CNS Neurosci Ther. 2023 May 11;29(10):3068–80. doi: 10.1111/cns.14249 (PMC10493658; doi:10.1111/cns.14249)
Supplement: Supplementary file 1 — Figure S1. [file CNS-29-3068-s001.pdf]

Supplementary Material S1 Original western blot details

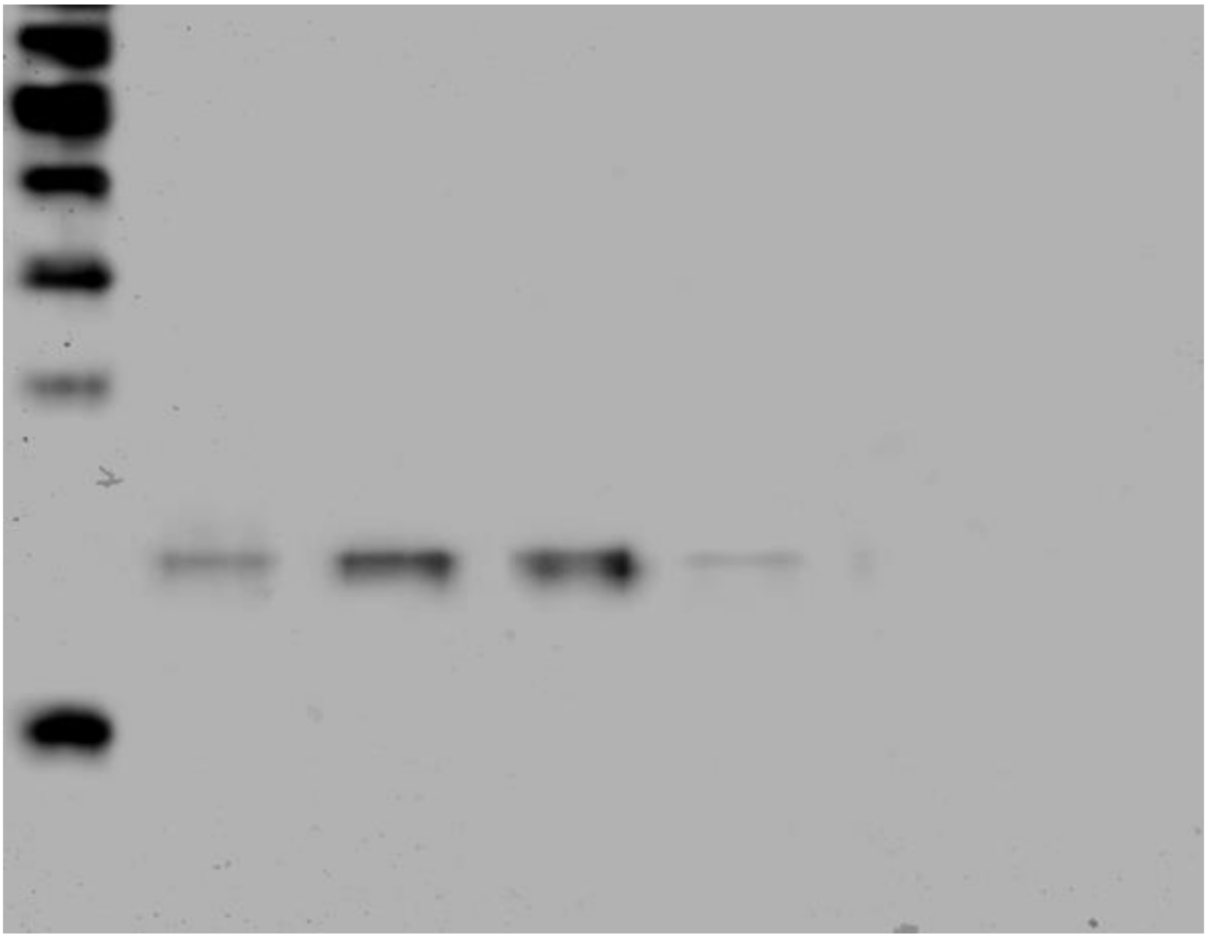

Figure S1.1. : The uncropped, untouched, full original image of western blot presenting BAX

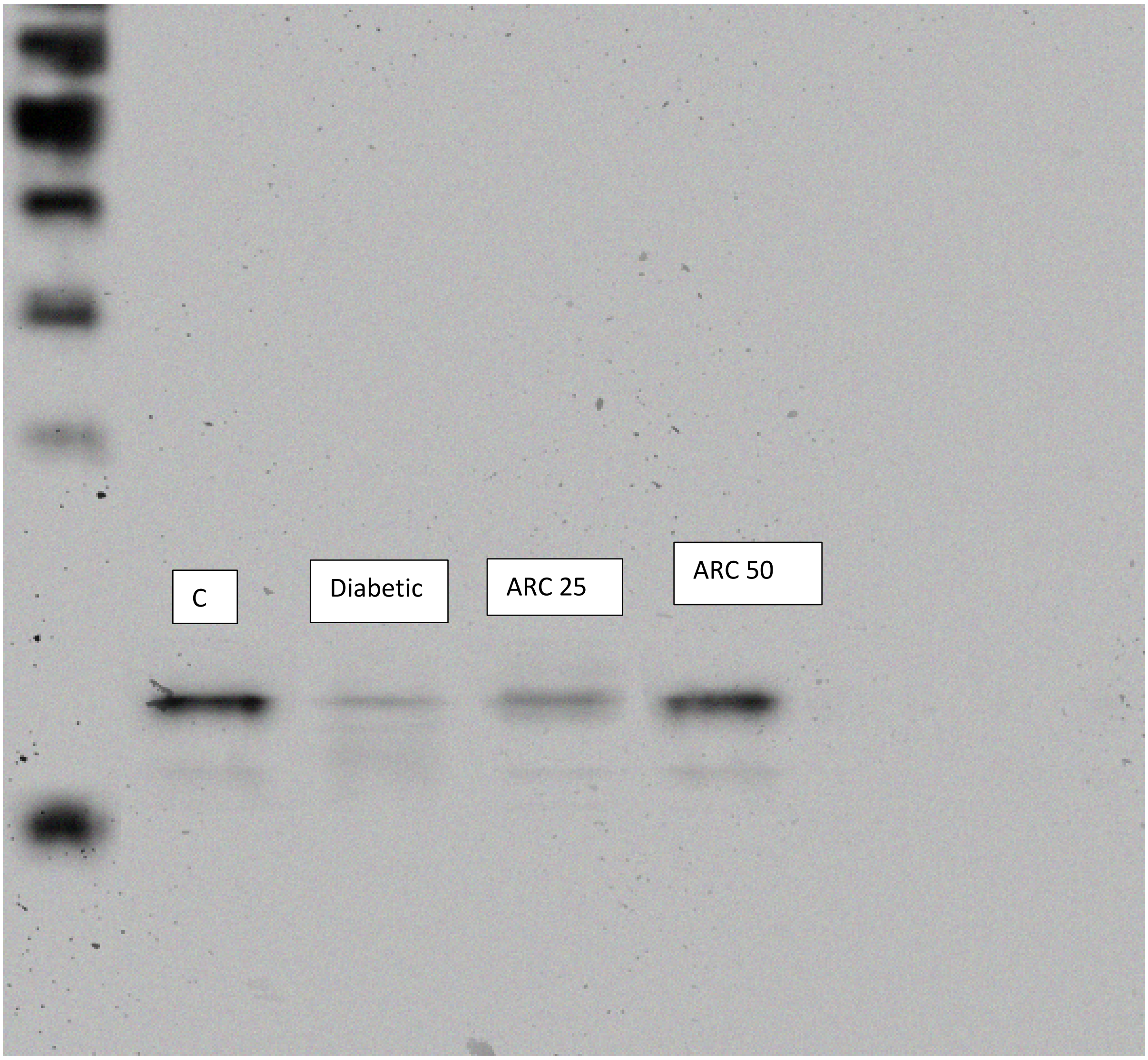

Figure S1.2. : The uncropped, untouched, full original image of western blot presenting BCL2

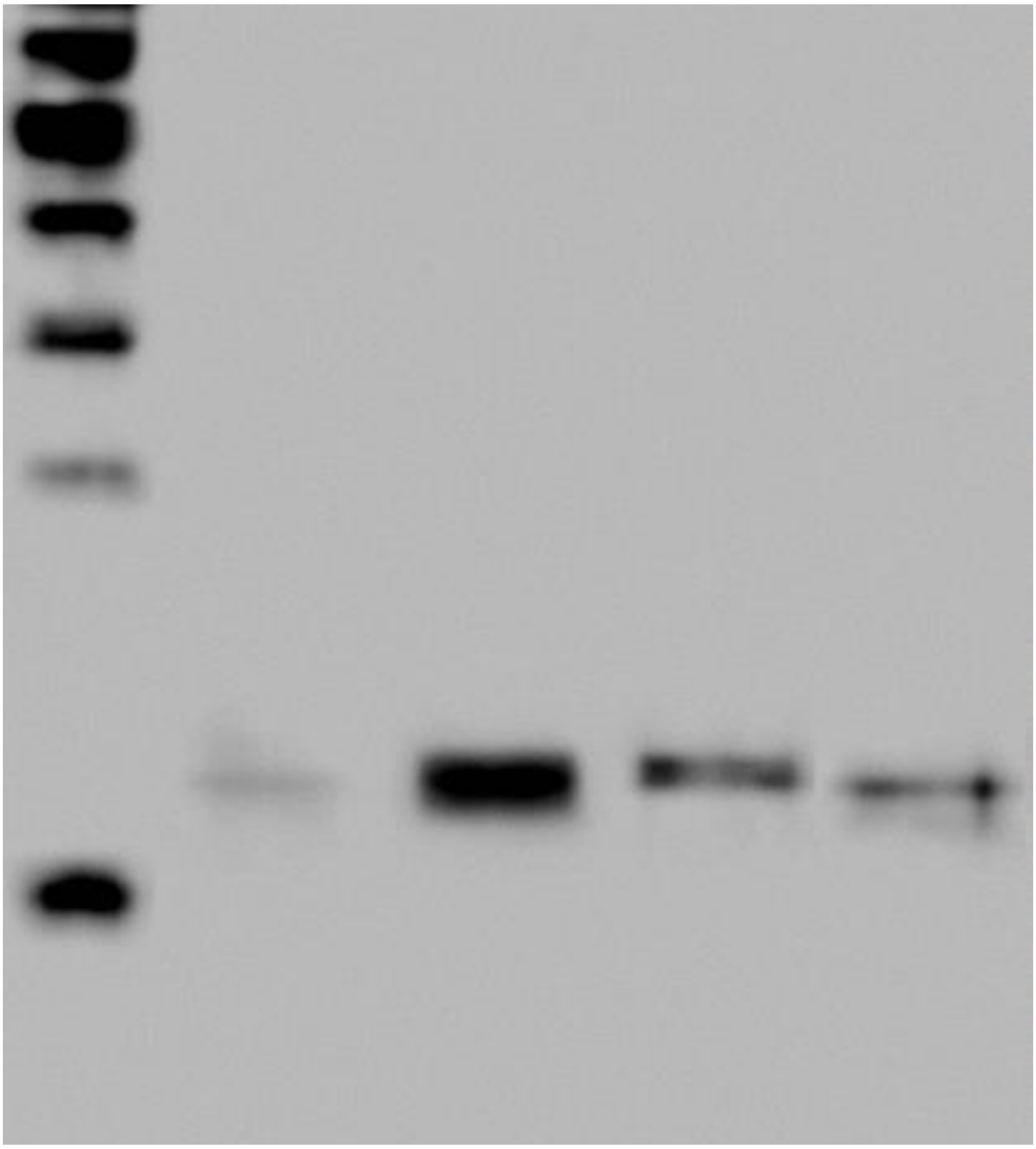

Figure S1.3. : The uncropped, untouched, full original image of western blot presenting cleaved caspase 3

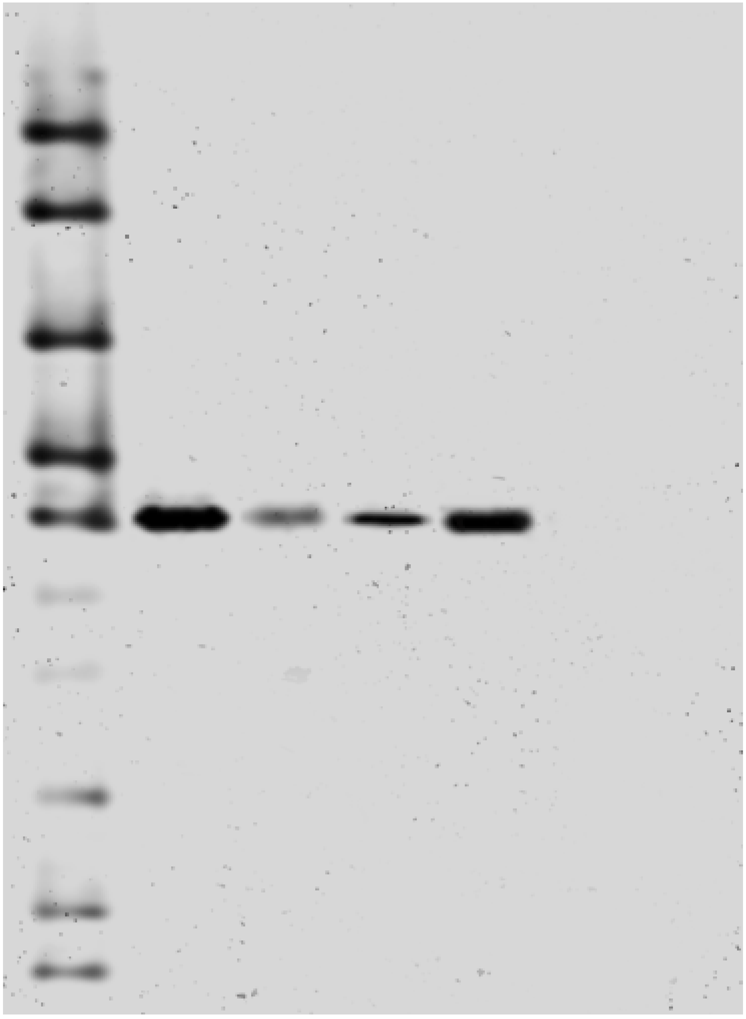

Figure S1.4. : The uncropped, untouched, full original image of western blot presenting Beclin

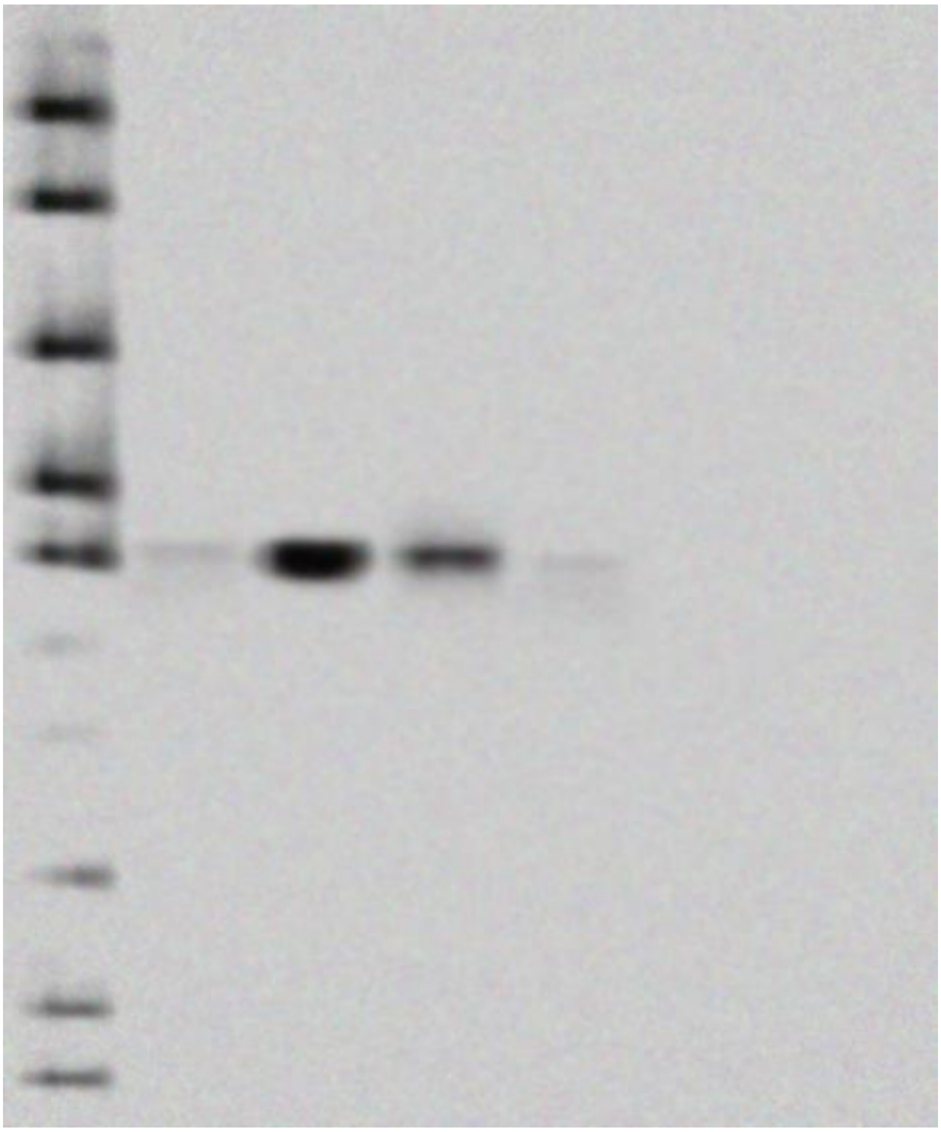

Figure S1.5. : The uncropped, untouched, full original image of western blot presenting p62

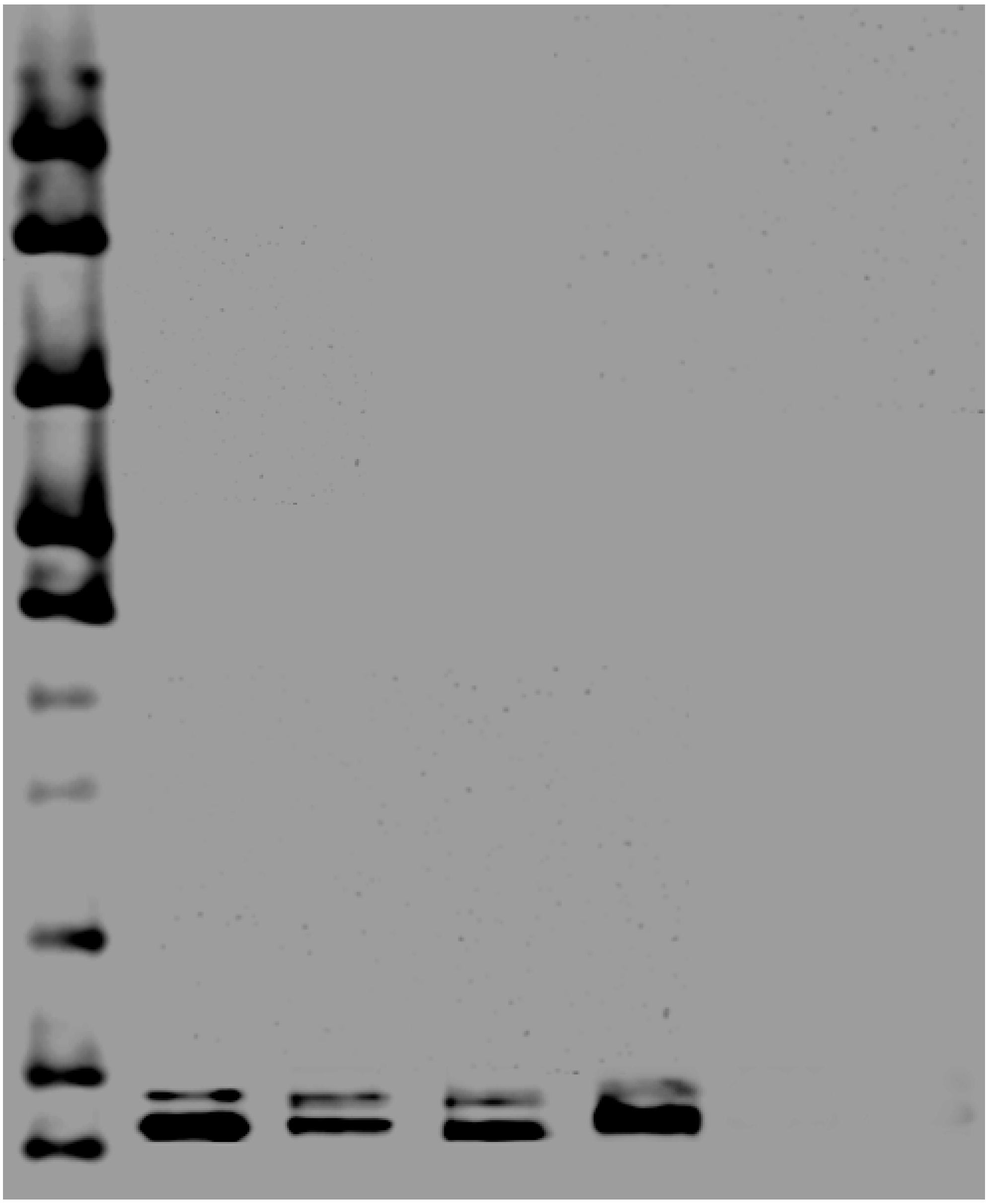

Figure S1.6. : The uncropped, untouched, full original image of western blot presenting LC3

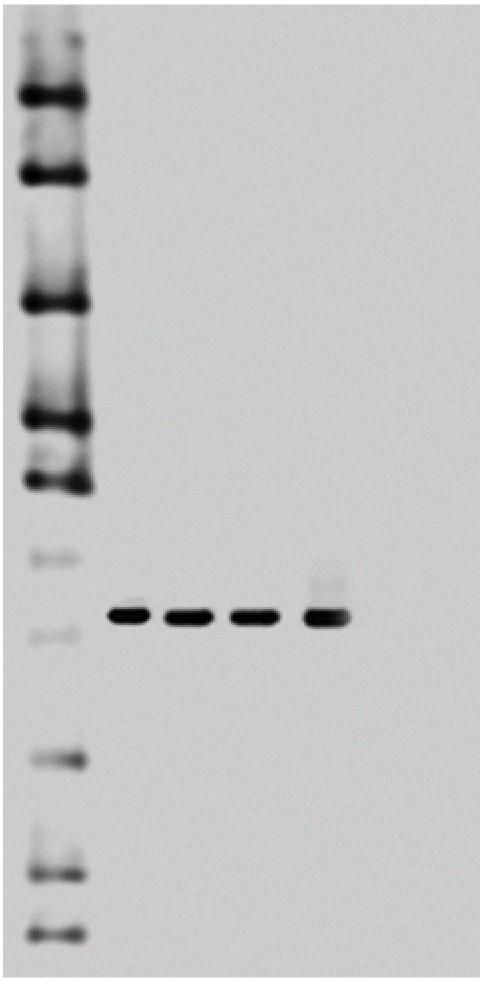

Figure S1.7. : The uncropped, untouched, full original image of western blot presenting B-actin

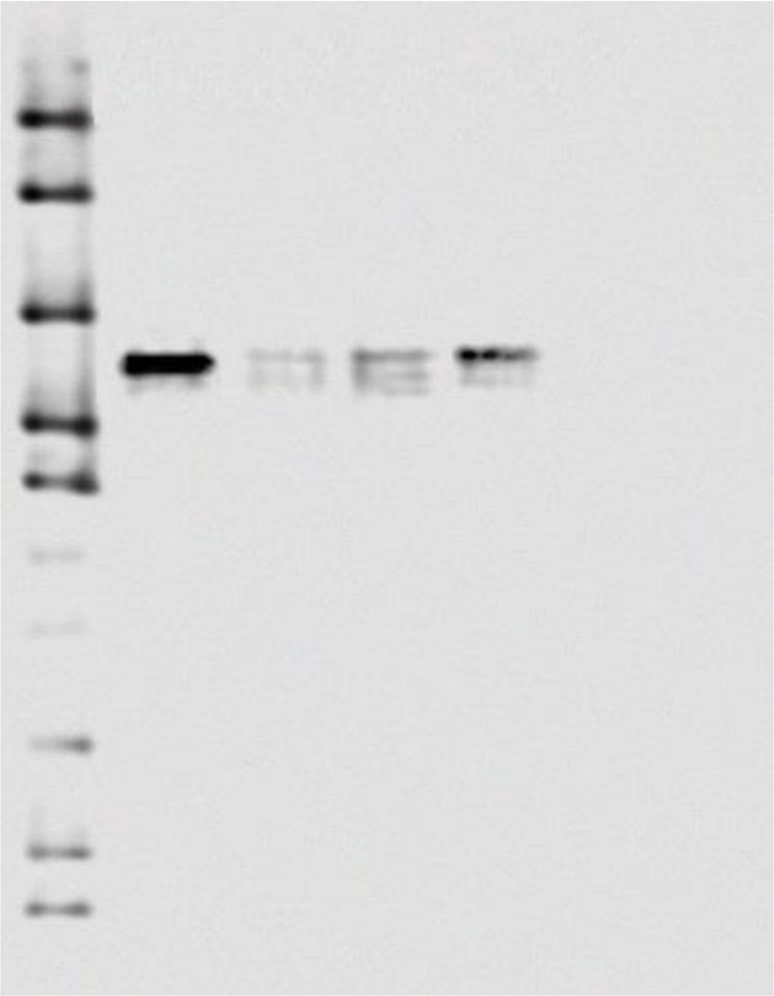

Figure S1.8. : The uncropped, untouched, full original image of western blot presenting AMPK
